# Compromised Effectiveness of Thermal Inactivation of *Legionella pneumophila* in Water Heater Sediments and Water, and Influence of the Presence of *Vermamoeba vermiformis*

**DOI:** 10.3390/microorganisms10020443

**Published:** 2022-02-15

**Authors:** Margot Cazals, Emilie Bédard, Margot Doberva, Sébastien Faucher, Michèle Prévost

**Affiliations:** 1 Department of Civil Engineering, Polytechnique Montréal, Montréal, QC H3C 3A7, Canada; emilie.bedard@polymtl.ca (E.B.); margot.doberva@polymtl.ca (M.D.); michele.prevost@polymtl.ca (M.P.); 2 Department of Natural Resource Sciences, Faculty of Agricultural and Environmental Sciences, McGill University, Sainte-Anne-de-Bellevue, QC H9X 3V9, Canada; sebastien.faucher2@mcgill.ca

**Keywords:** *Legionella pneumophila*, *Vermamoeba vermiformis*, hot water distribution system, water heater, thermal treatment, temperature, flow cytometry

## Abstract

Intermittent reduction of temperature set-points and periodic shutdowns of water heaters have been proposed to reduce energy consumption in buildings. However, the consequences of such measures on the occurrence and proliferation of *Legionella pneumophila* (*Lp*) in hot water systems have not been documented. The impact of single and repeated heat shocks was investigated using an environmental strain of *L. pneumophila* and a reference strain of *V. vermiformis*. Heat shocks at temperatures ranging from 50 °C to 70 °C were applied for 1 h and 4 h in water and water heaters loose deposits (sludge). The regrowth potential of heat-treated culturable *L. pneumophila* in presence of *V. vermiformis* in water heaters sludges was evaluated. A 2.5-log loss of culturability of *L. pneumophila* was observed in simulated drinking water at 60 °C while a 4-log reduction was reached in water heaters loose deposits. Persistence of *Lp* after 4 h at 55 °C was shown and the presence of *V. vermiformis* in water heater’s loose deposits resulted in a drastic amplification (5-log). Results show that thermal inactivation by heat shock is only efficient at elevated temperatures (50 °C) in both water and loose deposits. The few remaining organisms can rapidly proliferate during storage at lower temperature in the presence of hosts.

## 1. Introduction

*Legionella* reside in many aquatic environments and can develop in water systems from buildings, including those harbouring vulnerable populations such as healthcare facilities and long-term care facilities [1,2,3]. *L. pneumophila* causes most of the Legionnaires’ disease cases, a severe form of pneumonia associated with high morbidity for at risk populations such as the elderly or immunocompromised [3,4,5]. *L. pneumophila* is particularly adapted to hot water environments, with reported optimal temperatures for growth ranging between 25 °C and 45 °C, while temperatures of 50 °C and over have been shown to decrease the culturable *L. pneumophila* populations [3,5,6]. Optimal growth temperature as well as resistance to heat may vary significantly between strains of *L. pneumophila* studied. Temperature-resistant environmental strains have been isolated from hot water systems and from systems subjected to repeated thermal shocks [7,8,9]. For instance, Allegra et al. reported that 6 out of 12 clinical and environmental strains of *Legionella* maintained viability for 10% to 25% of the cell population after 30 min at 70 °C [10].

In Quebec, water heaters, as well as institutional, commercial or residential hot water distribution systems, are known as reservoirs involved in the dispersion of *Legionella* bacteria [8]. Indeed, it was reported that 33% of electrical water heaters were colonized by *Legionella*, with concentrations from 10 to more than 100 CFU/mL [11].

**Thermal control to prevent and remediate *Legionella* in hot water systems.** Maintaining temperature outside *Legionella*’s preferred growth range is the paramount and well demonstrated *Legionella* preventative control strategy in building water systems [3]. To prevent the risk of legionellosis, temperatures in water heaters, hot water distribution systems and associated devices are regulated in many countries, especially in healthcare facilities and retirement homes. Thermal control, often by maintaining temperatures over 55 °C across the hot water system, can be successful in controlling *Legionella* and *L. pneumophila* in hot water systems [3]. Longitudinal studies in hospital water systems have shown that increasing temperature at distal points from 50 °C to 55 °C significantly decreases positivity and concentrations of *Legionella* [12,13]. Temperature monitoring in hot water systems is considered a useful control tool to evaluate the risk of contamination by *Legionella* and more specifically *L. pneumophila* [1,14]. The Quebec construction code requires a minimal temperature of 60 °C to be maintained at the outlet of the water heater, and of at least 55 °C in the recirculation loop [15]. However, to prevent scalding in healthcare facilities and retirement homes, the temperature of hot water at the bathtub faucets and showers should not exceed 43 °C [15].

The efficiency of thermal control mainly relies on the maintenance of high temperatures at distal ends of the hot water distribution systems. A study with more than 30,000 water samples from 4600 German public buildings over 7 years revealed that mean temperatures measured at distal points were 7 °C and 11 °C lower than those measured in recirculation loops and flushed samples respectively, which corresponded to 10-fold higher concentrations of *Legionella* [16]. Efficient temperature control can only be achieved in hydraulically balanced systems without dead-end pipes and faulty devices. It can be difficult to reach target control temperatures at each outlet in large buildings where hydraulic balancing is not optimal [1,2,5,17]. Furthermore, temperatures in water heaters, reservoirs and building water systems vary in space and time as a function of system architecture, water demand, and hydraulics. In the distal part of the distribution system where recirculation is not present, temperatures oscillate between recirculation/post thermal mixing valve temperatures and room temperature between water uses. 

**Thermal shocks as an emergency response to *Legionella* contamination**. Curative heat shock treatment at 70 °C can be used to disinfect water system [3,5,18]. A temperature of 70 °C or more should be maintained for 20 to 30 min at each point of use [5,8]. Yet, sporadic thermal treatments have shown limited efficiency against *L. pneumophila* in time [9,19]. Similarly, thermal disinfection was conducted twice a day for two weeks by flushing hot water (60–70 °C) at taps and showers for several minutes in 4 buildings water systems in Finland. *Legionella* recolonized these systems within 4 months, despite maintaining the hot water system at 55 °C between and after thermal disinfection [20]. Therefore, thermal shocks are considered a temporary remedial or emergency response, not a preventive measure because of their limited impact over time, and their potential for the selection of heat resistant *L. pneumophila* strains [3]. More importantly, Allegra and colleagues showed that repeated heat shock treatments in a health care facility selected for heat resistance of *L. pneumophila* strains. *L. pneumophila* strains from a hot water circuit where temperature was frequently increased to 65 °C for a day were more heat resistant than those from a hot water system with sporadic heat treatments. Their results suggest that *Legionella* strains can adapt and become heat resistant after repeatedly applying super heat shock treatments [9]. To further support the short term benefits of shock heat treatment, it was shown that an elevated temperature set point at the water heater has a stronger impact on *Legionella* concentrations at distal taps than a one-time 30 min-heat shock at 60 °C [21]. More recently, Whiley and colleagues have hypothesized that superheat and flush thermal tolerant *Legionella* have a greater public health significance [18].

**Impact of hosts**. The regrowth of *Legionella* bacteria after heat treatment has been attributed to necrotrophic growth, their integration into biofilm, and their capacity to resist phagocytosis and grow within heat-resistant forms of protozoans [19,22,23,24]. Amoebae are the preferential *L. pneumophila* hosts in hot water systems [25,26]. Particularly, *L. pneumophila* can infect amoebae and even survive within them when they transform into cysts, which are known to be more resistant to disinfectants and temperature. For instance, Storey and colleagues showed that *L. pneumophila* can survive in *Acanthamoebae* cysts after 10 min at 80 °C [27]. *Vermamoeba vermiformis* are free-living amoebae ubiquitous in water environments, including in hospitals where they can survive to temperatures above 55 °C [28]. *V. vermiformis* is the most often associated host of *L. pneumophila* in warm- and hot-water distribution systems [29,30]. *L. pneumophila* residing in *V. vermiformis* cysts can resist temperatures from 50 °C to 70 °C [31,32]. Farhat et al. reported that cysts of *V. vermiformis* survive a heat shock of 70 °C for 30 min [33]. The survival of hosts is a critical factor for regrowth, as there is now a consensus that the control of eucaryotic hosts of *Legionella* is necessary to achieve effective *Legionella* control in water systems, as the ability of *Legionella* to grow without hosts is limited [3].

**Alternative temperature management for energy conservation.** In 2017, water heating represented 19.3% of the energy consumed in the average Canadian home [34]. In the context of energy conservation to reduce building’s carbon footprint, there is increased pressure to operate water heaters and building hot water systems at lower temperature set-points. However, lower temperature steady state operation increases the risk of *Legionella* in hot water heaters and distribution systems [3,16]. To reduce the energy consumption due to water heating, incentivized customer shutdowns of water heaters (WHs) have been applied to shave off peak demands. Thus, alternating cycles of elevated temperature and lower temperature operations during peak demand periods occur, and the high temperature cycles are analogue to heat shock treatments.

Electric water heaters are by design thermally stratified, unlike oil and gas water heaters and are therefore more susceptible to *Legionella* contamination [11,35,36,37,38,39]. This is attributed to the lower temperatures in the bottom of the water heater caused by thermal stratification and the presence of sediments. Indeed, sediments accumulate in the conical bottom sections of electric WHs, and potentially provide nutrients and growth-promoting conditions for multiplication of *L. pneumophila* and host cells and turn these devices into preferential niches for *Legionella* bacteria [11,35].

The main objective of this study was to investigate the effects of single and repeated 1 h- and 4 h-heat treatments at temperatures from 50 to 70 °C on the survival and growth of *V. vermiformis* and an environmental strain of *L. pneumophila* isolated from a health care facility faucet biofilm [40]. The impact of repeated short-term exposures to higher temperatures followed by stagnation at lower temperatures was investigated to simulate conditions in hot water systems submitted to curative heat shock and water heater peak demand shutdowns. The dynamics of *V. vermiformis* and *Legionella* decay and growth were investigated in water and in water heater loose sediments (sludge) to evaluate the survival in the different phases present in water heaters and hot water systems. Water heater loose deposits were preferred to the liquid phase because more hosts were expected, and the lower temperature encountered at the bottom of electric stratified water heaters would make these deposits a more susceptible niche for *L. pneumophila* to grow.

This study aims to provide evidence to support the risk assessment of periodical shutdowns of water heaters in order to allow electric demand peak shaving for energy conservation, while avoiding unintended consequences on water safety.

## 2. Materials and Methods

### 2.1. Preparation of Inocula

**Amoeba** Culture of the ATCC 50237^TM^ strain of *Vermamoeba vermiformis* was performed using Falcon^®^ T75 cell-culture vented flasks (Fisher Scientific, ON, Canada) with 25 mL of modified PYNFH media incubated at 30 °C. *V. vermiformis* trophozoites and cysts were enumerated using a haemocytometer. A volume of 100 µL was mixed with 300 µL of clean modified PYNFH media and 100 µL of 0.4% methylene blue, and 10 µL of this mix were put on the haemocytometer. The cells were then enumerated using an Olympus BX51 microscope using 100× magnification. 

Flow cytometry was also used to enumerate amoebae and to differentiate viable and dead cells. All samples were diluted by a factor of 10, and each staining method was performed in duplicates with 300 µL of sample and 3 µL of SYBRGreen I (SG, 100× concentrated) or a mix of SYBRGreen and Propidium iodide (SGPI, end concentration of 6 µM of PI). Before the addition of the dye, the samples were incubated for 3 min at 37 °C. Once the plate was ready with samples and dyes, it was incubated in the dark for 10 min at 37 °C. The flow cytometry assay was then conducted using a BD Accuri-C6 flow cytometer and data were analysed with the Accuri sampler software (BD Biosciences, NJ, United States of America). Flow cytometer readings were conducted using FL1 (530–533 nm) and FL3 (>670 nm) filters. The FL1 threshold was set at 130,000 and the SSC threshold at 90,000.

For the flow cytometry data analysis, the sample heated at 100 °C was considered as the control for dead *V. vermiformis* and its graph of the red fluorescence (FL3) in function of the green fluorescence (FL1) was used to define the gate gathering all the dead amoebae in the SGPI-stained samples. Then, the same cytograms of FL3 vs FL1 were used to estimate the number of dead *V. vermiformis* (events in the gate) in SGPI-stained samples. For each temperature, the mean value of the percentage of dead cells of the duplicates was calculated.

***Legionella*** An environmental strain of *Legionella pneumophila* serogroup 5 (CEAEQ isolate ID Q076826-03) was extracted from the biofilm of a faucet from a healthcare facility [40]. The conservation and culture methods were those described in Bédard et al., 2021 [41]. Briefly, after storage at −80 °C in 60% glycerol, it was cultured for 3 days at 36 °C on buffered charcoal yeast extract (BCYE, Oxoid, ON, Canada). Resulting colonies were directly inoculated into sterile yeast extract broth with the Oxoid growth supplement SR0110 (Oxoid, ON, Canada) and incubated at 36 °C for 18 h. Suspensions were centrifuged at 3000× *g* for 30 min to harvest the cells, which were then washed twice with sterile water (simulated drinking water or 0.22 µm filtered water from water heaters, as presented in Section 2.3) and resuspended in the same medium at 1 × 10^8^ cells/mL. Cells were starved for 5 to 10 days at room temperature.

### 2.2. Collection and Concentration of Water Heaters’ Loose Deposits

Sediments were collected from two water heaters located in two different municipalities (A and B). The 2 water heaters were completely emptied, then partially refilled to resuspend all the deposits accumulated in the bottom. The water heaters were connected to a sediment trap system to collect the resuspended deposits. With this system, between 30 and 40 L were collected from each water heater. The deposits were then concentrated. For the water heater A, successive volumes of 250 mL of the mix of water and deposits were centrifuged 15 min at 1500× *g*, to recover 400 mL of sediments. The sediments were then equally distributed in 10 bottles and resuspended in a final volume of 500 mL with filtered water heater’s water. For the water heater B, an additional step was required due to the presence of very light sediments that were not captured by centrifugation. Following centrifugation, supernatant was filtered on 0.45 µm to recover fine particles. Deposits’ characterisation is provided in Table S1.

### 2.3. Temperature Inactivation Testing

**Temperature inactivation of *V. vermiformis*.** Cells from two volumes of 25 mL of *V. vermiformis* culture were washed three times as follows: initial cultures were centrifuged 10 min at 200× *g*, the supernatant was then discarded, and the pelleted amoebae were resuspended in 25 mL of autoclaved tap water filtered on 0.22 µm for a final concentration of 1 × 10^5^ cells/mL. The temperature assay was conducted on 5 mL of *V. vermiformis* suspension at 1 × 10^5^ cells/mL. A total of 8 temperatures were tested with a contact time of 4 h: 25 °C, 30 °C, 37 °C, 40 °C, 43 °C, 50 °C, 55 °C and 60 °C. A test was also conducted at 100 °C and was considered as the control for dead amoebas.

**Temperature inactivation of *L. pneumophila* in water (hot water distribution system conditions).** Starved *L. pneumophila* cells were resuspended in 50 mL sterile tubes containing 20 mL of simulated drinking water (final concentration = 1 × 10^8^ cells/mL). Simulated drinking water and starvation were performed as described in Bédard et al., 2021 [41]. Suspensions were treated for 1 h at 55 °C, 60 °C or 70 °C. After 1 h of thermal treatment, tubes were stored at 36.5 ± 0.5 °C in the dark and samples were taken for enumeration after 1 h, 6 h, 1 day, 2 days, 3 days, 1 week, 2 weeks and a month. A temperature of 36.5 °C was chosen as it is representative of the temperature encountered in temperature-controlled faucets like electronic faucets and thermostatic mixing valves [42,43]. Negative (without *L. pneumophila*) and positive controls (*L. pneumophila* without thermal treatment, left in the dark at 36.5 ± 0.5 °C for the duration of the experiment) were also monitored. *L. pneumophila* concentrations were estimated by culture on BCYE agar, with an incubation in the dark at 36.5 ± 0.5 °C for 7 days, with a first enumeration after 3 days.

**Temperature inactivation of *L. pneumophila* in water heater loose deposits.** The short term (1 to 24 h) impact of the thermal stress on *L. pneumophila* was also assessed in the loose deposits collected from two water heaters located in two different municipalities (Table S1). Both loose deposits were free of *L. pneumophila* when sampled (tested with the Legiolert enzymatic test (IDEXX, ME, United States of America)) and no hosts were observed by microscopy. Duplicate volumes of 18 mL of homogenised sludge were heated at the desired temperature and then inoculated with 2 mL of *L. pneumophila* suspension for a final concentration of 10^6^ CFU/mL. Testing was conducted at six temperatures (40, 45, 50, 55, 60 and 65 °C) for 5 different durations (1 h, 2 h, 4 h, 6 h and 24 h). Negative control samples were monitored at t = 0 h and t = 24 h to evaluate the natural decay of *L. pneumophila* concentration without any thermal stress. The Legiolert enzymatic test was used to quantify culturable *L. pneumophila* using the 1 mL non-drinking water protocol and dilutions of the loose deposits.

To assess the impact of a daily exposure of 4 h at the target temperature within the water heater, repeated 4 h exposures at 55 °C followed by 20 h at 40 °C were performed on water heater sludges inoculated with *L. pneumophila.* Inactivation of *L. pneumophila* after repeated exposure to heat was determined using duplicates of 100 mL of each sludge inoculated with a final concentration of 1 × 10^6^ cells/mL and left in the dark at 40 °C for 72 h. Then, samples were exposed to a temperature of 55 °C for 4 h and then to 40 °C for 20 h. Deposit samples were then exposed daily to 55 °C for 4 h, for 5 heating cycles in total. Between cycles, samples were kept in the dark at 40 °C. Samples were taken before each heating cycle to quantify the culturable *L. pneumophila* concentration using the Legiolert enzymatic test 1 mL non-drinking water protocol following the manufacturer’s instructions.

**Temperature inactivation of *L. pneumophila* in water heater loose deposits in the presence of *V. vermiformis*.** For this assay, 100 mL of each loose deposits containing 1 × 10^6^ cells/mL of *L. pneumophila* were treated for 4 h at 40 °C, 55 °C and 60 °C. Following treatment, for each suspension and each temperature, samples were divided in four identical volumes of 2 mL. The first two volumes were spiked with *V. vermiformis* to a final concentration of 1 × 10^6^ cells/mL and the two remaining volumes were kept as controls without amoeba. Tubes were then incubated in the dark at 36.5 ± 0.5 °C for 4 days and 7 days, and the concentrations of culturable *L. pneumophila* were measured using the enzymatic Legiolert test.

## 3. Results

### 3.1. Thermal Inactivation of L. pneumophila and V. vermiformis in Hot Water

#### 3.1.1. Impact of a Short Duration Heat Shock on *L. pneumophila* in Water

**Figure 1:**
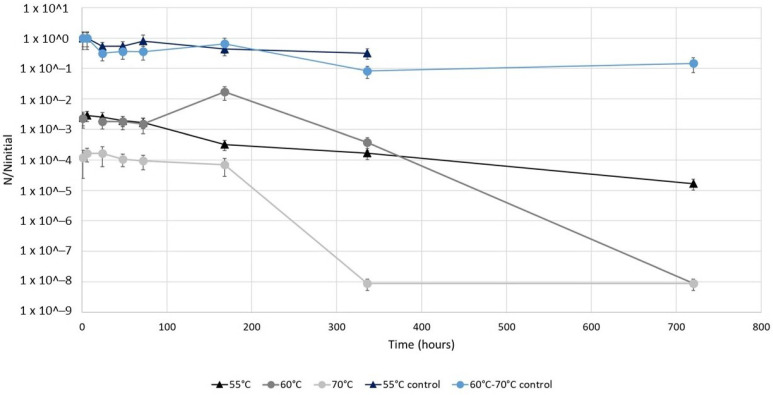
Temporal evolution of the ratio of the concentrations of culturable *L. pneumophila* in samples after a 1 h heat treatment at 55 °C, 60 °C or 70 °C on the initial concentrations of culturable *L. pneumophila* in respective controls left at 36.5 ± 0.5 °C. Experiments were conducted on an environmental *L. pneumophila* strain previously starved in simulated drinking water at room temperature. After the 1 h heat shock, bacteria were stored at 36.5 ± 0.5 °C for up to 1 month.

The aim of the first assay was to evaluate the impact of a short duration (1 h) heat shock (55 °C, 60 °C and 70 °C) applied to *L. pneumophila* before its storage at a temperature of 36.5 ± 0.5 °C for up to a month. This combination simulates the conditions faced by bacteria during a short heat shock in a dead-end of a building water system: high temperature in the water network for 1 h, then the temperature comes back to its previous value (Figure 1).

*L. pneumophila* concentrations in control suspensions left at 36.5 ± 0.5 °C during the experiment decreased by one log only after 336 h (for exact values, see Table S3). The first point of each curve represents the initial impact of the 1 h heat shock at each temperature after just 1 h of storage. Thus, the heat shocks at 55 °C and 60 °C resulted in an immediate 2.5 log reduction in the culturable *L. pneumophila* populations, while at 70 °C the culturable *L. pneumophila* population was reduced by 4 logs. Then, for the first 168 h (7 days) of storage, culturable *L. pneumophila* suspensions remained constant, except for the sample at 60 °C for which an increase of a log was observed after 168 h. Finally, after 168 h of storage, the concentrations of culturable *L. pneumophila* decreased in all the heat-treated samples, with an absence of these bacteria reached after 720 h (5-log reduction) and 336 h (4-log reduction) of storage at 60 °C and 70 °C, respectively. After a 1 h treatment at 55 °C, a reduction of only 2 logs was reached at the end of the experiment (720 h of storage).

#### 3.1.2. Impact of Elevated Temperatures on the Form and Decay of *V. vermiformis* in Hot Water

This assay aimed to evaluate the survival of *V. vermiformis* in water at temperatures representative of different parts of drinking water and hot water networks. The survival of amoebae *V. vermiformis* was evaluated by exposing them to different temperatures for 4 h, followed by their enumeration by microscopy and flow cytometry. 

**Figure 2:**
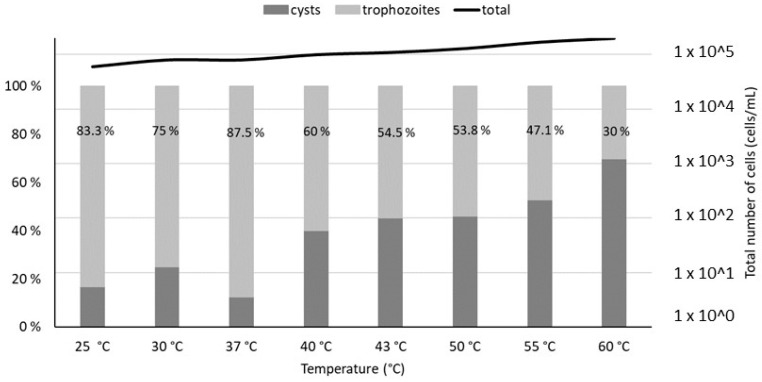
Trophozoites and cysts percentages estimated by optical microscopy (100× magnification), after a 4 h thermal treatment of *V. vermiformis* suspensions at temperatures from 25 °C to 60 °C. The percentages of trophozoites in the total *V. vermiformis* population are indicated on the graph. The line represents the total population of amoebae in each sample.

Microscopic measurements revealed a relatively constant total population of *V. vermiformis* (between 2 × 10^4^ and 2 × 10^5^ cells/mL, exact values given in Table S2) with minimal lysis. However, a shift between the trophozoite and cysts forms of *V. vermiformis* is observed at temperatures above 40 °C, with cysts becoming predominant at 55 °C (Figure 2)

**Figure 3:**
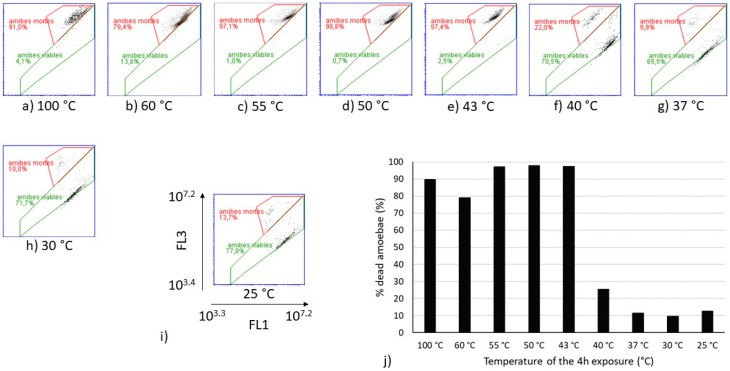
Cytograms of heat-treated *V. vermiformis* samples with SYBRGreen I and Propidium iodide (SGPI) staining (**a**–**h**), and evolution of the percentage of dead *V. vermiformis* depending on the temperature of the 4 h treatment (**j**). (**i**) is a zoom on one of the cytograms with the specification of the scales of the axes. The gate in red dot lines delimited the population of dead amoebae. “amibes mortes” means dead amoebae and “amibes viables” means viable amoebae. For the percentages indicated on the cytograms, a comma separates the decimals.

Flow cytometry analyses were performed on the same heat-treated *V. vermiformis* suspensions (Figure 3). The cytograms obtained for temperatures from 25 °C to 40 °C show similar partitioning between the viable and dead amoebae, as well as those obtained from 43 °C to 60 °C. The sample heated at 100 °C was considered as the dead control. Because the only population obtained from 43 °C to 60 °C on FL1-FL3 cytograms seemed equivalent to the one observed at 100 °C, it can be assumed that this population is representative of the dead amoebae *V. vermiformis*. The proportion of dead amoebae estimated by flow cytometry on Figure 3 shows a drastic shift between 40 °C and 43 °C. Indeed, the increase of 3 °C induced a transfer from 25% of dead *V. vermiformis* to more than 90% of dead amoebae.

### 3.2. Thermal Inactivation of L. pneumophila in Loose Deposits of Water Heaters

Different contact times, as well as repeated short heat exposures, were performed on a hot water system-adapted *L. pneumophila* strain to evaluate its survival in water heater sludge.

#### 3.2.1. Heat Exposures of Different Durations

Most of the microorganisms present in electric water heaters are found in the loose deposits at the bottom of the water heater, a niche providing particles for attachment, nutrients, and lower temperatures. To investigate the impact of a thermal treatment on *L. pneumophila* in resuspended loose deposits of electric water heaters, heat treatments of up to 24 h were conducted on two samples of resuspended water heater loose deposits inoculated with 1 × 10^6^ CFU/mL of a starved environmental strain of *L. pneumophila*.

**Figure 4:**
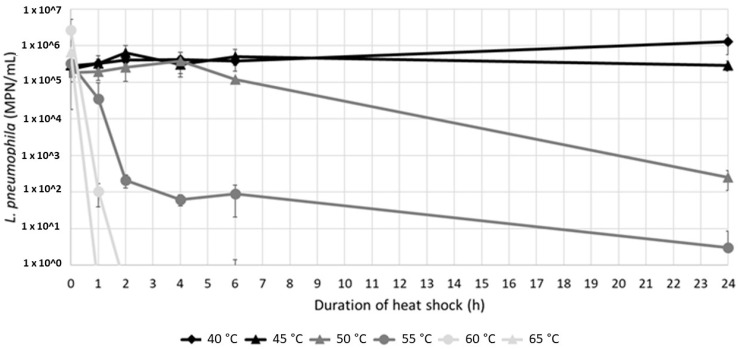
*L. pneumophila* concentrations (mean value of sludges A and B, *n* = 4) depending on the contact time and temperature. The detection limit of the enzymatic test is 1 MPN/mL.

Culturable *L. pneumophila* were detected in the resuspended sludges at various levels (from 2.49 × 10^2^ to 1.27 × 10^6^ MPN/100 mL) after a 24 h exposure to temperatures between 40 °C and 55 °C (Figure 4). A 3-log reduction was achieved at 50 °C with a long exposure of 24 h, while the culturable population remained constant at 45 °C, and showed an almost 1-log increase at 40 °C. On the other hand, after 24 h at 55 °C a few bacteria were still detected. At 60 °C and 65 °C, short two- and one-hour contact times were sufficient to completely abate the culturable population.

Two different inactivation kinetics were observed at 50 °C and 55 °C. At 50 °C, no inactivation was observed in the first 4 h, followed by a progressive decrease after 4 h (Figure 4). On the contrary, at 55 °C, culturable *L. pneumophila* rapidly decreased after a contact time of 2 h, followed by a slower rate of decrease.

#### 3.2.2. Impact of Repeated Short Heat Shocks

**Figure 5:**
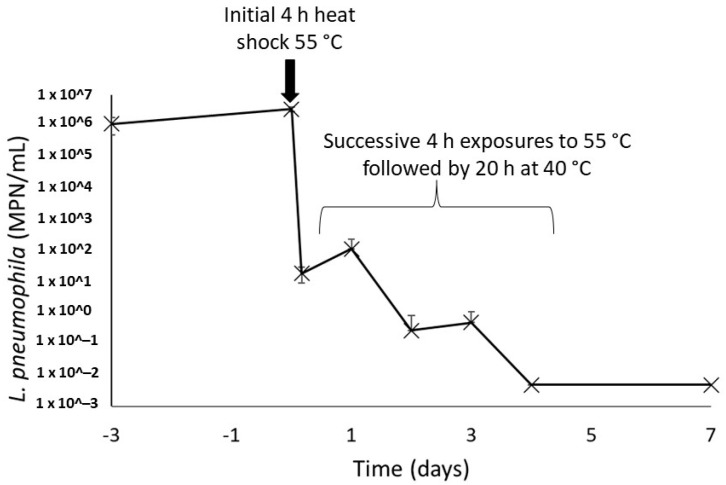
Concentrations of culturable *L. pneumophila* in water heater sludges after repeated 4 h thermal shocks at 55 °C. The concentrations on days 4 and 7 were under the detection limit of the enzymatic test in all samples, duplicate of each water heater loose deposits.

Once present within the recirculating hot water system, bacteria undergo periodic heat exposures every time they flow through the water heater. This was simulated by repeating the exposure to heat assay performed on the environmental strain of *L. pneumophila.* In this assay, a drastic decrease of 5 logs of the culturable *L. pneumophila* population after the initial 4 h heat shock at 55 °C was observed (Figure 5). The following four heat shocks gradually further reduced the culturability of this population, and no more culturable *L. pneumophila* could be found on day 4 in both sludges. A final measurement performed 72 h after the final heat shock showed no reactivation of the bacteria population left for 20 h at a favourable temperature of 40 °C after heat shock.

### 3.3. Resuscitation of L. pneumophila by V. vermiformis in Water Heater Loose Deposits

**Figure 6:**
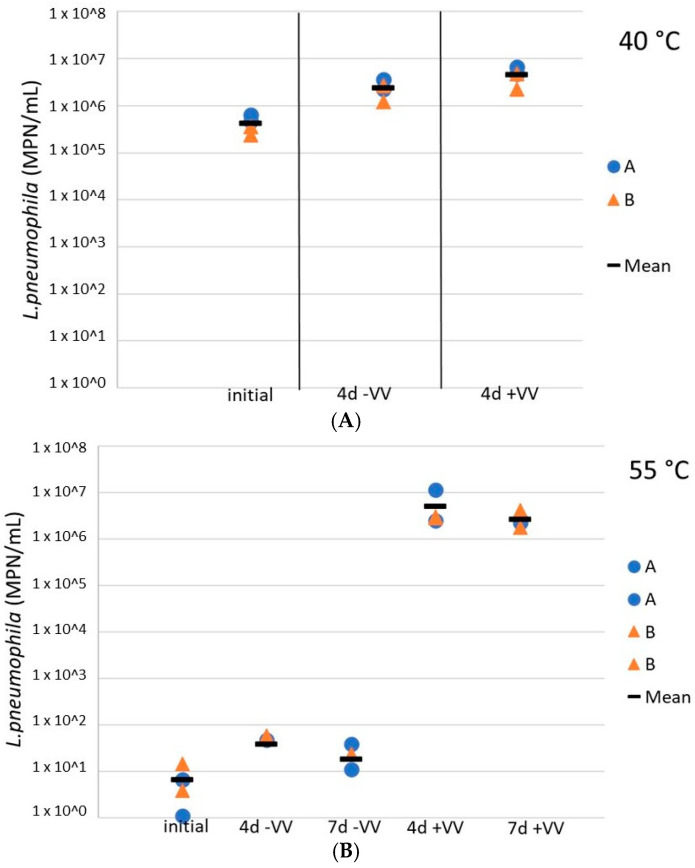
Concentrations of culturable *L. pneumophila* during storage at 37 °C in water heater loose deposits subsequent to a thermal shock of 4 h at 40 °C (**A**) and 55 °C (**B**), with and without the presence of amoebae *V. vermiformis*. Blue dots are for sludge A samples, orange triangles represent results from sludge B samples, and black horizontal lines illustrate the mean values of all samples. +VV = sludges inoculated with *V. vermiformis*, −VV = sludges free of *V. vermiformis*.

Finally, *L. pneumophila* cells were exposed to *V. vermiformis* following temperature inactivation to assess the potential for regrowth downstream in the distribution system, in the presence of hosts. Here, the role of amoebae in the regrowth of *L. pneumophila* after a heat shock was evaluated in resuspended water heater loose deposits. The addition of *V. vermiformis* 4 days after a 4 h exposure at 40 °C did not impact the culturable *L. pneumophila* concentrations (difference of less than 0.5 log), the latest remained relatively constant at more than 1 × 10^6^ cells/mL (Figure 6A). However, at 55 °C, although a small increase of less than a log was observed for culturable *L. pneumophila* concentrations 4 days after the heat shock, *L. pneumophila* concentrations in sludges inoculated with *V. vermiformis* increased by 5 logs as compared to sludges without hosts, so there was an important regrowth in the presence of amoebae (Figure 6B). Moreover, the concentration remained constant for the following 72 h. At 60 °C the culturable *L. pneumophila* concentrations remained null with or without hosts (data not shown).

## 4. Discussion

### 4.1. Thermal Heat Shock Inactivation of L. pneumophila and V. vermiformis in Water Heaters and Hot Water Systems

Curative heat shock treatments at temperatures of 60–75 °C have been used by many healthcare facilities as temporary curative actions in response to elevated concentrations of *Legionella*. spp. or *L. pneumophila* [3,5]. Notably, the effectiveness of shock heat treatment is limited in time and recolonization of the treated systems is often observed [5,44]. 

In this study, we found that a 1 h heat exposure led to an immediate 4-log reduction of culturable *L. pneumophila* at 70 °C and 2.5-log reduction at 55 °C and 60 °C in simulated drinking water. Following the 1 h exposure at 60 °C and 70 °C, complete loss of culturability was reached when left stagnant at 36.5 °C after 1 month and 2 weeks respectively. Indeed, after the initial decrease, concentrations of culturable *L. pneumophila* remained constant for the first 168 h (1 week) of storage, before starting to decrease again. The constant phase following the first decrease may be due to the necrotrophic ability of *L. pneumophila* [24]. In comparison, a 1 h exposure at 65 °C led to a reduction in culturable *L. pneumophila* greater than 6 logs in resuspended loose water heater deposits, while 4-log and 1.5-log reductions were reached after a 1 h exposure at 60 °C and 55 °C respectively in the same type of sample. These results suggest that some *L. pneumophila* can survive a thermal shock of 30 min at 70 °C in water and sludge and can persist in a water heater or a hot water network and could then seed distal parts of the distribution systems.

**Resistance of *V. vermiformis* to heat.** A rapid shift from a predominantly trophozoite form to a predominantly cyst form is observed between 50 °C and 55 °C as shown by microscopy. Flow cytometry confirms a shift from viable to dead amoebae occurring at lower temperatures between 40 °C and 43 °C. These results suggest an elevated susceptibility to temperatures greater than 43 °C associated with a significant loss of membrane integrity as determined by PI staining. The reference strain used in this study (*Hartmannella vermiformis* Page–ATCC 50237) was isolated from the drain of a hospital cooling tower. Cooling tower water temperatures typically do not exceed 48 °C. Our observations are in agreement with a previous study showing that *V. vermiformis* in biofilms were 2-log lower at 42 °C than at 38 °C and that the lack of a thermotolerant host at the highest temperature may prevent the proliferation of *L. pneumophila* in the studied system [6]. The results also concurred with the hypothesis that thermotolerance is strain dependent. Thus, depending on the origin of the strain of *V. vermiformis* used for the assays, studies showed absence of *V. vermiformis* in biofilms at 41 °C [6] while others reported 1-log and 2-log reductions after an exposure at 50 °C for 30 min [31] and 60 min [32].

The resistance to heat of amoebae can be attributed to the strain intrinsic resistance, the form in which it is present (trophozoite or cyst) and the method to assess its persistence. Indeed, amoebae have 2 stages in their life cycle: the mobile form of trophozoite, and a dormant form emerging under prolonged unfavourable conditions, the cyst [45]. The cysts are known to be more resistant to numerous disinfectants and to temperature [31,45]. However, depending on the enumeration technique used and the strain studied, reported levels of heat resistance of *V. vermiformis* vary. Thus, studies using sequencing or qPCR to enumerate *V. vermiformis* after heat treatment [33] or thermal stresses [46], and studies based on culture [26,32] reported various heat resistance.

**Detection and quantification of *V. vermiformis*.** Although the presence of hosts in a water system is considered as the determining factor for the proliferation of *L. pneumophila*, the methods available for the detection of protozoan hosts are cumbersome. In this study, flow cytometry was used to enumerate amoebae. Flow cytometry (FC) presents numerous advantages over the standard culture and microscopy methods. First, it provides a faster response than culture, as the results can be obtained hours after sampling. Moreover, a significant advantage when dealing with environmental samples, detection of amoebae by culture is difficult to achieve while it is more convenient using flow cytometry. The staining and flow cytometry protocols used in this study for the monitoring of *V. vermiformis* were adapted from protocols for the differentiation and enumeration of viable and dead bacteria in water and biofilm samples [47]. Flow cytometry has been previously used to study amoebae, especially to follow the encystation process and to differentiate viable and dead cells [48,49]. However, this study is, to our knowledge, the first to adapt an FC-staining procedure for the quantification of bacteria to the enumeration of amoebae in water samples. The results obtained showed that the staining and FC protocols used allowed the differentiation of viable and dead amoebae in water. Still, further investigations are needed to confirm which life cycle state of *V. vermiformis* compose each population observed on the cytograms, and to assess whether the gates defined here can be accurately applied in environmental samples where higher densities of other microorganisms are present.

### 4.2. Thermal Inactivation of L. pneumophila in Water and Hot Water Loose Deposits

The resistance of the environmental strain isolated from a healthcare facility in Québec was assessed in two types of loose deposits. The strain appears to be well adapted to warm water temperatures considering the absence of inactivation at 40 °C and 45 °C, as compared to the inactivation constants reported in previous studies [50,51], and its persistence at 50 °C and 55 °C. Inactivation kinetic constants were calculated for each temperature based on the inactivation curves of the 24 h exposure of *L. pneumophila* in water heater’s loose deposits, and were compared to those obtained in previous studies (Table 1). The graphical method proposed by Körmendy and Körmendy (1997) was applied [52]. The inactivation of *L. pneumophila* at 55 °C does not follow a simple first order kinetics as proposed in the Bigelow’s method [52] (data shown in Figure S1). Moreover, for all the temperatures tested, the inactivation kinetic constants were lower than those reported by Sanden et al. and Stout et al., somehow indicating that the current tested strain is more resistant to heat than those tested by these authors [50,51] or is more acclimatized to its environment prior to the application of heat stress.

Heat resistance of *L. pneumophila* has been shown to vary between strains [3] and environmental strains of *L. pneumophila* isolated from hot water systems can develop a greater resistance to temperature, even above 60 °C [7,10]. During heat treatment experiments conducted on 4 *L. pneumophila* strains (2 references and 2 environmental isolated from hot tap water), Cervero-Aragó et al. noted significant differences between the inactivation patterns by culture depending on the strain and the temperature applied (50 °C, 55 °C, 60 °C, 65 °C and 70 °C) [53]. Similarly, Allegra et al. obtained different thermal inactivation curves for 12 *Legionella* strains at 70 °C using flow cytometry [10]. Indeed, after 30 min at 70 °C, the authors were still able to detect between 10% and 25% of viable but nonculturable (VBNC) cells of 6 of the 12 tested strains of *Legionella*. 

The general resistance of *Legionella* to temperature is typically assessed in laboratory experiments using suspensions of cultured *Legionella* exposed to a constant temperature. However, the results of such assays and the resulting resistance to temperature are influenced by the state of the bacteria. Indeed, it was shown that starving bacteria prior to temperature exposure can increase their resistance to environmental stress [54]. Thus, Chang and colleagues showed that the heat disinfection efficiency on *L. pneumophila* cells starved for 1 to 2 months decreased, revealing the resistance of long-term starved *L. pneumophila* against thermal disinfection [54]. It must be noted that the strain used in this study was washed and acclimatized to low nutrient conditions before exposure to heat. This can be one of the factors explaining the lower inactivation kinetic constants as well as the resistance to temperature up to 55 °C found in the present work.

**Implications for dynamic hot water systems.***Legionella* and their hosts present in recirculating hot water distribution systems undergo repeated heat exposures associated with:

(1) passage through the water heater and reservoirs, before returning in the hot water distribution system;
(2) storage in the water heater or reservoirs during periods of low or no use. Temperature conditions will vary depending on the water heater type, hot water demand and stratification. For instance, thermal stratification typically results in 10 to 15 °C lower temperatures in the bottom part of the reservoir of electric water heaters;
(3) exposure in the recirculation loops. In a well-balanced system, a temperature of more than 55 °C should be maintained.

When water heaters are operated dynamically to conserve energy during peak demand periods, *Legionella* will be exposed to lower temperatures during shutdown periods followed by periods of elevated temperatures during normal usage. Our experimental results of heat shock simulations show that, when heated at 60 °C in presence of water heater loose deposits, it took between one and two hours for suspended *L. pneumophila* to completely lose cultivability, while less than an hour was required at 65 °C. Thus, a 4 h-exposure to temperatures equal or higher than 60 °C could inactivate *Legionella* that is present in the water. 

Our results also provide some insight in the potential benefits from repeated cycles of elevated temperature to provide a barrier for the proliferation of *Legionella* in water and sludge of water heaters. The repetition of 4 h exposures at 55 °C on water heaters sludges inoculated with *L. pneumophila* followed by 20 h at 40 °C simulated night-time temperature exposure followed by lower temperatures during the day. Results revealed an absence of culturable *L. pneumophila* after 3 cycles of 4 h exposure at 55 °C, and no regrowth was observed after 72 h at 40 °C. Thus, comparatively to single exposures where culturable *L. pneumophila* were still detected after 24 h at 55 °C, repetitive heating appeared more effective against the studied environmental strain.

It should be noted that this experiment was conducted in laboratory conditions and not in a real water heater, and that no measurement was made on the medium term (several weeks) to verify if repeated heating was still efficient or if the strain was able to adapt. On the other hand, a 4 h-exposure at 60 °C and 65 °C was sufficient to eliminate all culturable *L. pneumophila* in water heaters’ loose deposits, suggesting that fixing the regulated temperature of water heaters to 60 °C would efficiently prevent *L. pneumophila* proliferation in these devices if the temperature is also reached at the bottom of the water tank. These observations are in accordance with a study where the hot water temperature at the water heater outlet was increased from 55 to 60 °C. They reported a gradual decrease of *L. pneumophila* concentrations and positivity over 18 months [13]. Rhoads and colleagues also showed that the temperature setting (from 39 °C to 58 °C) of continuously recirculating water heaters was a crucial factor for the prevention of *L. pneumophila* growth in recirculating water lines and distal faucets [46].

### 4.3. Resuscitation of L. pneumophila by V. vermiformis after a 4 h-Heat Treatment in Water Heater Loose Deposits

The co-cultivation of heated *L. pneumophila* with *V. vermiformis* in water heater sludges clearly highlighted the role hosts can play in reviving *L. pneumophila* after thermal treatments. As other *Legionella* species, *L. pneumophila* can survive phagocytosis and multiply in many protozoans like ciliates and amoebae [55]. Integration and replication of *L. pneumophila* in *V. vermiformis* has already been observed in several studies [56,57].

Here, 4 days after a 4 h treatment at 55 °C, *L. pneumophila* concentrations were 5-log higher in presence of *V. vermiformis* than in the absence of amoeba, and the concentrations remained stable for 3 more days. However, the presence of amoebae was not sufficient to increase the concentrations of *L. pneumophila* previously treated at 60 °C for 4 h, either by resuscitation or growth of survivors. These findings are similar to those of Cervero-Aragó et al. who reported that the association amoeba-bacteria between *Acanthamoeba* spp. and *L. pneumophila* had the most detrimental impact on the effectiveness of thermal treatments at 50 °C and 55 °C compared to the control without amoeba [53]. 

VBNC *L. pneumophila* can invade and replicate in amoebae, leading to the resuscitation of these bacteria and the release of more viable bacteria in the immediate environment [58,59]. In fact, protocols have been defined to resuscitate VBNC *Legionella* cells via the co-culture with amoebae [60]. However, when *L. pneumophila* is exposed to higher temperatures, cells are no longer viable and cannot be resuscitated by the presence of hosts. Results from Cervero-Aragó et al. support this, since the presence of a host did not increase the exposure time required to achieve 4-log reduction at higher temperatures (60 °C, 65 °C and 70 °C) [53]. In addition to helping *Legionella* bacteria to survive heat treatments, the presence of amoebae also influences the resistance to disinfection and the infectivity of these bacteria. Indeed, it was underlined by several studies that *L. pneumophila* were more resistant to antibiotics and other disinfectants after their passage through amoebae and were also more infectious [61,62]. Epalle et al. showed that heat-treated environmental *L. pneumophila* resuscitated in *A. polyphaga* were able to infect U937 and HL-60 macrophage-like cells while they could not infect these same cells right after the heat treatment at 70 °C for 30 min [44]. The authors underlined that amoebae present in hot water systems may facilitate the resuscitation of VBNC *L. pneumophila* induced by thermal treatments and may possibly lead to outbreaks [44].

Because of the key role played by protozoans in the persistence of *L. pneumophila* in water distribution systems, and in its resistance to many remediation strategies, it should be a priority to better understand the impact of disinfectants and temperature on all the microorganisms associated with *L. pneumophila* in water systems. This is especially the case for protozoan hosts, as host control appears to be the best approach to tackle more effectively the proliferation of *L. pneumophila* in such systems [3,63,64].

### 4.4. Limitations of this Study

To our knowledge this work is the first to monitor the impact of temperature on *L. pneumophila* in loose deposits collected from the bottom of water heaters and in hot water, and to evaluate the influence of the presence of a host after thermal treatments. Nevertheless, only a single *L. pneumophila* environmental strain was tested, and it would be interesting to investigate the fate of other strains in similar conditions. Moreover, in this study *L. pneumophila* were added to the water and the sludges samples which originally did not contain any *Legionella* bacteria. The microbial diversity present at first in the loose deposits may affect the survival of *L. pneumophila* in these samples during the study and the results of similar assays conducted on different deposits samples may lead to different conclusions. Moreover, biofilms and hosts present in plumbing can help protect *L. pneumophila* against temperature, but these factors were not present in our lab experiment. 

In this study, only culturable *L. pneumophila* were monitored, using culture on BCYE media and an enzymatic test. However, several studies using other techniques such as flow cytometry mentioned the presence of viable but non culturable (VBNC) *Legionella* bacteria after heat treatments. For instance, VBNC *L. pneumophila* of 3 different strains (one reference, one clinical and one environmental) were still detected by flow cytometry after 30 min at 60 °C and 70 °C [44]. Moreover, even is culture is considered the best indicator of risk of infection, the loss of culturability does not necessarily equate to the loss of infectivity. Indeed, it was shown that VBNC *L. pneumophila* cells produced by heat exposure at 55 °C, 60 °C and 70 °C were able to infect THP-1 macrophages and *Acanthamoeba castellanii* cells for 85 days at 55 °C and 60 °C, and 8 days at 70 °C [65].

Finally, only one strain of amoeba was used in this study. Thus, different results may be obtained with another strain or another genus of amoebae if used in a thermal inactivation assay. Indeed, Cervero-Aragó and colleagues showed that an exposure of 30 min at 50 °C of a lab strain of *V. vermiformis* induced a reduction of more than 2 logs of its trophozoite form and a reduction of less than 1 log of its cyst form, while the reduction of cysts of an environmental strain of *V. vermiformis* subjected to the same test was higher than 1 log [31]. The authors conducted the same assay on a lab and an environmental strain of *Acanthamoeba* and reported a higher decrease of the concentrations of their trophozoites (3- log reductions) but similar reductions of their cysts concentrations [31]. Other studies reported reductions of 1 log of cysts of *Acanthamoeba castellanii* after 10 min at 55 °C (lab strain) [27], and a reduction of 5 logs after 60 min at 60 °C (clinical isolate) [66]. Thus, it may be interesting to repeat a similar assay as the one described in the present study with other hosts of *L. pneumophila* found in water systems to assess their thermal resistance and the risk they may represent in the context of energy saving measures.

### 4.5. Considerations for the Operation of Residential Water Heaters and Hot Water Distribution Systems

The results of the present study provide additional evidence that reducing the set point temperature or sporadic shutdown of water heaters in buildings to conserve energy should be subjected to a risk analysis to carefully establish its benefits. Reducing water usage and lowering hot water temperature may promote the proliferation of opportunistic premise plumbing pathogens, such as *Legionella* [4,11].

Domestic water heaters have been shown to be frequently positive for *Legionella*, especially in the loose deposits found at their bottom, and positivity has been clearly correlated with temperature. *L. pneumophila* was detected in loose sediments of 45% of devices set at temperatures lower than 40 °C, while it was detected in only 14% of devices set at higher temperatures [11]. Water heater shutdown to accommodate peak demand periods should be implemented with compensating measures, such as increased base line temperature and improved design, to minimize thermal stratification and the accumulation of loose deposits during normal operations. 

In residential and large building hot water systems, sporadic lower temperature conditions are likely to increase the potential for *Legionella* growth and the associated exposure risk for the occupants, especially in buildings housing at-risk populations such as healthcare facilities, long term care and retirement homes containing aerosol-producing devices. To reconcile energy conservation and infection prevention, measures should be taken to limit the additional risk associated with these periods of vulnerability. Optimizing the operation of the water heater and water system can be achieved through multiple actions such as improving hydraulics, maintaining optimal thermal regime, increasing temperature set-points, and adding on-site disinfection. 

## 5. Conclusions

- Exposure of an environmental strain of *L. pneumophila* to 60 °C for 1 h led to a 2.5-log to 4-log loss of culturability in simulated drinking water and resuspended water heater loose deposits.
- Exposure to temperatures over 43 °C decreases the viability of *V. vermiformis* and causes a rapid shift to the dead form.
- Successive daily exposures at 55 °C for 4 h can prevent the growth of culturable *L. pneumophila* after one week.
- After a 4 h exposure to 55 °C, *L. pneumophila* concentrations with *V. vermiformis* are 5 log higher than without host 4 days after the treatment, and concentrations remain constant for at least 3 more days.
- The relevance of meeting regulated, and recommended water heaters and hot water system minimal set temperatures were confirmed.

## Figures and Tables

**Table 1:** Inactivation kinetic constants of *L. pneumophila* for temperatures between 40 °C and 60 °C. Sanden et al., 1989 [50] is for a strain of *L. pneumophila* serogroup 1 isolated from cooling tower water, inactivation measured in chlorine-free water. Stout et al., 1986 [51] is for 3 *L. pneumophila* strain of serogroup 5, inactivation measured in Buffered Yeast Extract Broth (BYEB).

Temperature and Time Lapse	k in this Study (h^−1^)	k in Previous Studies
From 0 to 24 h, 40 °C	0 *	
From 0 to 24 h, 45 °C	0 *	k = 0.024 h^−1^ [50]
From 0 to 4 h, 50 °C	0 *	k = 0.16 h^−1^ [50]
From 4 to 24 h, 50 °C	0.16	
From 0 to 2 h, 55 °C	1.6	k = 4.3 h^−1^ (measured on 20 min) [50]
From 2 to 24 h, 55 °C	0.034	
From 0 to 1 h, 60 °C	4.27	k = 81 h^−1^ [50] k from 17.6 to 26 h^−1^ (measured on 20 min) [51]

* These constants were considered null because there was no inactivation.

## Supplementary Materials

The following are available online at https://www.mdpi.com/article/10.3390/microorganisms10020443/s1, Figure S1: Results of the Kormendy and Kormendy graphical method, Table S1: Characteristics of the water and the deposits on the two water heaters studied, Table S2: Total populations of *V. vermiformis* estimated by microscopy after a 4 h-exposure at temperatures from 25 °C to 60 °C, Table S3: Mean values and standard deviations of the control samples for the assay on the long-term efficiency of 1 h heat exposures of *Legionella pneumophila*.
