# Supplementary material for: Compromised Effectiveness of Thermal Inactivation of Legionella pneumophila in Water Heater Sediments and Water, and Influence of the Presence of Vermamoeba vermiformis
Source: Microorganisms. 2022 Feb 15;10(2):443. doi: 10.3390/microorganisms10020443 (PMC8874534; doi:10.3390/microorganisms10020443)
Supplement: Supplementary file 1 [file microorganisms-10-00443-s001.zip › Cazals2022_SuppMat/Cazals2022_TableS1.pdf]

Table S. 1: Characteristics of the water and the deposits on the two water heaters studied

Parameters and units	Water Heater A	Water Heater B
	Water	
Chlorine residual (mgCl2/L) [1]	≤ DL	≤ DL
Total chlorine (mgCl2/L)	≤ DL	≤ DL
pH	8.0	8.2
Alkalinity (mgCaCO3/L)	27	180
Calcium (mgCaCO3/L) [2]	47	131
Total hardness (mgCaCO3/L)	53	239
Temperature in the bottom of the water heater during sampling (°C)	33.8	37.5
Total dissolved solids (mg/L)	96	376
Dissolved O2 (mg/L)	10	2
Conductivity (µS/cm²) [2]	150	587
	Deposits	
Turbidity (NTU)	142	342
Suspended matter (mg/L)	1344	2275
Volatile suspended solids (mg/L)	367	189
% Volatile solids	25	8
Magnesium (mg/L)	5.9	15.6
Aluminum (µg/L)	4.0	3.5
Calcium (mg/L)	13.5	30.1
Iron (µg/L)	249.9	558.7
Manganese (µg/L)	18.2	148.6
Copper (µg/L)	4.0	1196
Aerobic heterotrophic bacteria (CFU/mL)	415 000	40 000
L. pneumophila (MPN/mL)	ND	ND

[1] DL = 0,05 mg Cl_2/L; [2] measured in municipal treated water
