# Supplementary material for: Compromised Effectiveness of Thermal Inactivation of Legionella pneumophila in Water Heater Sediments and Water, and Influence of the Presence of Vermamoeba vermiformis
Source: Microorganisms. 2022 Feb 15;10(2):443. doi: 10.3390/microorganisms10020443 (PMC8874534; doi:10.3390/microorganisms10020443)
Supplement: Supplementary file 1 [file microorganisms-10-00443-s001.zip › Cazals2022_SuppMat/Cazals2022_TableS2.pdf]

Table S. 2: Total populations of *V. vermiformis* estimated by microscopy after a 4h-exposure at temperatures from 25°C to 60°C.

Temperature of the 4h exposure	Total population of *V. vermiformis* (cells/mL)
25°C	6×10^4
30°C	8×10^4
37°C	8×10^4
40°C	1×10^5
43°C	1.1×10^5
50°C	1.3×10^5
55°C	1.7×10^5
60°C	2×10^5
