# Supplementary material for: Compromised Effectiveness of Thermal Inactivation of Legionella pneumophila in Water Heater Sediments and Water, and Influence of the Presence of Vermamoeba vermiformis
Source: Microorganisms. 2022 Feb 15;10(2):443. doi: 10.3390/microorganisms10020443 (PMC8874534; doi:10.3390/microorganisms10020443)
Supplement: Supplementary file 1 [file microorganisms-10-00443-s001.zip › Cazals2022_SuppMat/Cazals2022_TableS3.pdf]

Table S. 3: Mean values and standard deviations of the control samples for the assay on the long-term efficiency of 1h heat exposures of *Legionella pneumophila*

Time after the 1h heat exposition (hours)	Control 55°C - Mean (CFU/mL)	Control 55°C – Standard Deviation		Control 60°C and 70°C - Mean (CFU/mL)	Control 60°C and 70°C – Standard Deviation	
1	5.00E+08	1.73E+08	(34.64%)	5.67E+07	2.31E+07	(40.75%)
6	5.00E+08	1.73E+08	(34.64%)	5.67E+07	2.31E+07	(40.75%)
24	2.67E+08	5.77E+06	(2.17%)	1.77E+07	2.08E+06	(11.78%)
48	2.70E+08	5.29E+07	(19.60%)	2.07E+07	4.16E+06	(20.15%)
72	4.00E+08	1.73E+08	(43.30%)	2.03E+07	4.73E+06	(23.24%)
168	2.17E+08	4.16E+07	(19.22%)	3.67E+07	1.15E+07	(31.49%)
336	1.60E+08	2.00E+07	(12.50%)	4.67E+06	5.77E+05	(12.37%)
720	/ [1]	/	/	8.33E+06	2.52E+6	(30.20%)

[1] No value available for the control sample of the 55°C assay after 720 hours.
