# Supplementary figures and images for: Compromised Effectiveness of Thermal Inactivation of Legionella pneumophila in Water Heater Sediments and Water, and Influence of the Presence of Vermamoeba vermiformis
Source: Microorganisms. 2022 Feb 15;10(2):443. doi: 10.3390/microorganisms10020443 (PMC8874534; doi:10.3390/microorganisms10020443)

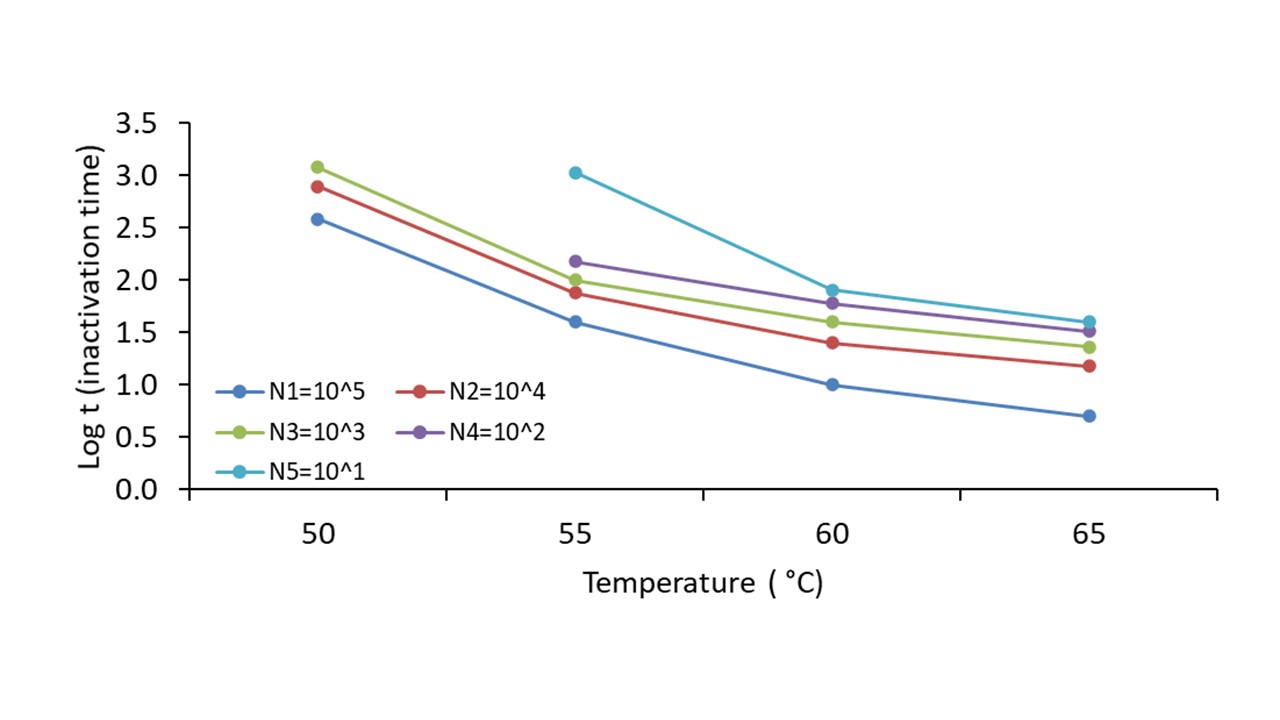

Supplement: Supplementary file 1 [file microorganisms-10-00443-s001.zip › Cazals2022_SuppMat/Cazals2022_FigureS1.jpg]
